# The gut microbiota during the progression of atherosclerosis in the perimenopausal period shows specific compositional changes and significant correlations with circulating lipid metabolites

**DOI:** 10.1080/19490976.2021.1880220

**Published:** 2021-03-11

**Authors:** Qinghai Meng, Menghua Ma, Weiwei Zhang, Yunhui Bi, Peng Cheng, Xichao Yu, Yu Fu, Ying Chao, Tingting Ji, Jun Li, Qi Chen, Qichun Zhang, Yu Li, Jinjun Shan, Huimin Bian

**Affiliations:** a School of Pharmacy, Nanjing University of Chinese Medicine, Nanjing, China; b Jiangsu Key Laboratory for Pharmacology and Safety Evaluation of Chinese Materia Medica, School of Pharmacy, Nanjing University of Chinese Medicine, Nanjing, China; c School of Medicine and Life Sciences, Nanjing University of Chinese Medicine, Nanjing, China; d First School of Clinical Medicine, Nanjing University of Chinese Medicine, Nanjing, China

**Keywords:** Atherosclerosis, menopause, gut microbiota, lipid metabolomics, ApoE ^−/-^ mice

## Abstract

Atherosclerosis (AS) is exacerbated in the perimenopausal period, which significantly increases the incidence rate of cardiovascular disease. The disruption of the gut microbiota has been associated with AS or menopause, but the specific changes of AS-associated gut microbiota in the perimenopausal period remain largely unknown. As lipid abnormalities are mainly responsible for AS, the relationship between lipid metabolism abnormalities and gut microbiota disruptions during menopause is rarely reported hitherto. In the present study, ApoE^−/-^ mice fed with a high-fat diet (HFD) were subjected to ovariectomy and supplemented with estrogen. The ovariectomized HFD-fed ApoE^−/-^ mice underwent significant AS damage, hepatic lipid damage, hyperlipidemia, and changes of lipid metabolism- and transport-related enzymes. There was significantly higher abundance of some lipid metabolites in the plasma of ovariectomized HFD-fed ApoE^−/-^ mice than in non-ovariectomized ones, including cholesterol esters, triglycerides, phospholipids, and other types of lipids (free fatty acids, acylcarnitine, sphingomyelins, and ceramides). The administration of estrogen significantly reduced the contents of most lipid metabolites. The diversity and composition of gut microbiota evidently changed in ovariectomized HFD-fed ApoE^−/-^ mice, compared to HFD-fed ApoE^−/-^ mice without ovariectomy. In contrast, with estrogen supplementation, the diversity and composition of gut microbiota were restored to approach that of non-ovariectomized HFD-fed ApoE^−/-^ mice, and the relative abundances of some bacteria were even like those of C57BL/6 mice fed with a normal diet. On the other hand, the transplantation of feces from C57BL/6 mice fed with normal diet to ovariectomized HFD-fed ApoE^−/-^ mice was sufficient to correct the hyperlipidemia and AS damage, and to reverse the characteristics changing of lipid metabolomics in ovariectomized HFD-fed ApoE^−/-^ mice. These phenomena were also been observed after transplantation of feces from estrogen-treated ovariectomized HFD-fed ApoE^−/-^ mice to ovariectomized HFD-fed ApoE^−/-^ mice. Moreover, the gut microbiota and lipid metabolites were significantly correlated, demonstrating that the changes of serum lipids may be associated with the gut microbiota disruptions in the perimenopausal period. In conclusion, the gut microbiota during the progression of AS in the perimenopausal period showed specific compositional changes and significant correlations with circulating lipid metabolites. Estrogen supplementation may exert beneficial effects on gut bacteria and lipid metabolism.

## 1. Introduction

During menopause, females undergo down-regulation of estrogen and dysfunction of hormone receptors, also being prone to various diseases including cardiovascular disease (CVD).^1,2^ Age-specific analysis of clinical data suggests that the risk factors for coronary atherosclerotic heart disease in women increase along with age.^3^ Large-scale population studies have verified the associations of age and menopause with the lipid levels and CVD in women.^4,5^

In women with sufficient estrogen, the gut microbiota presents species diversity, and beneficial bacteria are dominant, inhibiting the growth of harmful bacteria and autotoxicity.^6,7^ The relative abundances of beneficial bacteria such as *Lactobacillus* and *Bifidobacteria* significantly reduce in females with perimenopausal syndrome, and those of harmful bacteria such as *Enterobacter* soar increase.^8,9^ In addition to menopause, the gut microbiota also dominantly participates in the progression of obesity,^10,11^ diabetes^10^, and atherosclerosis.^12^

Santos-Marcos JA et al. analyzed the differences in gut microbiota in premenopausal and postmenopausal women. Their results showed that the ratio of *Firmzicute*s/*Bacteroides* in the gut microbiota was higher, the relative abundances of *Lachnospira* and *Roseburia* were higher, and the relative abundances of *Prevotella, Parabacteroides* and *Bilophila* were lower in postmenopausal women.^9^ Choi et al. compared the gut microbiota characteristics of diet-induced and bilaterally ovariectomized obese mice. They had similar gut microbiota compositions, but with differences in *Bifidobacterium animalis, Dorea, Akkermansia muciniphila* and *Desulfovibrio*.^13^ Therefore, the gut microbiota may undergo specific compositional changes during the perimenopausal period.

As a major risk factor for CVD,^14,15^ AS is characterized by abnormal lipid metabolism, leading to cholesterol deposition on the arterial wall and eventually forming plaques.^16^ It is well documented that hypercholesterolemia was a direct cause and an independent risk factor for AS.^17–20^ Reducing the plasma cholesterol level plays a key role in preventing and treating AS.^21^ In the past decade, the imbalance of the gut microbiota has been closely related to the progression of AS.^12^ However, the relationship between the specific compositional changes of the gut microbiota and the changes of circulating lipid metabolites during the progression of AS in the perimenopausal period remains unclear. Meanwhile, whether estrogen insufficiency in this period is a key factor promoting AS progression still needs in-depth studies.

Herein, bilaterally ovariectomized ApoE^−/-^ mice were used to reveal the specific compositional changes of the gut microbiota during perimenopause. Plasma lipid metabolites and the fecal gut microbiota were detected, and the correlations between them were analyzed in the present study. We provided some evidence that AS was aggravated and the gut microbiota specifically changed in bilaterally ovariectomized ApoE^−/-^ mice. Besides, plasma lipid metabolites were markedly disturbed and significantly associated with gut microbiota changes. Estrogen deficiency may dominate in the changes of the gut microbiota and plasma lipid metabolites in the perimenopausal period, and accelerate the progression of AS.

## 2. Results

### 2.1. Estrogen supplementation reduced the acceleration of atherosclerosis caused by ovariectomy in HFD-fed ApoE^−/-^ mice

**Figure 1.**
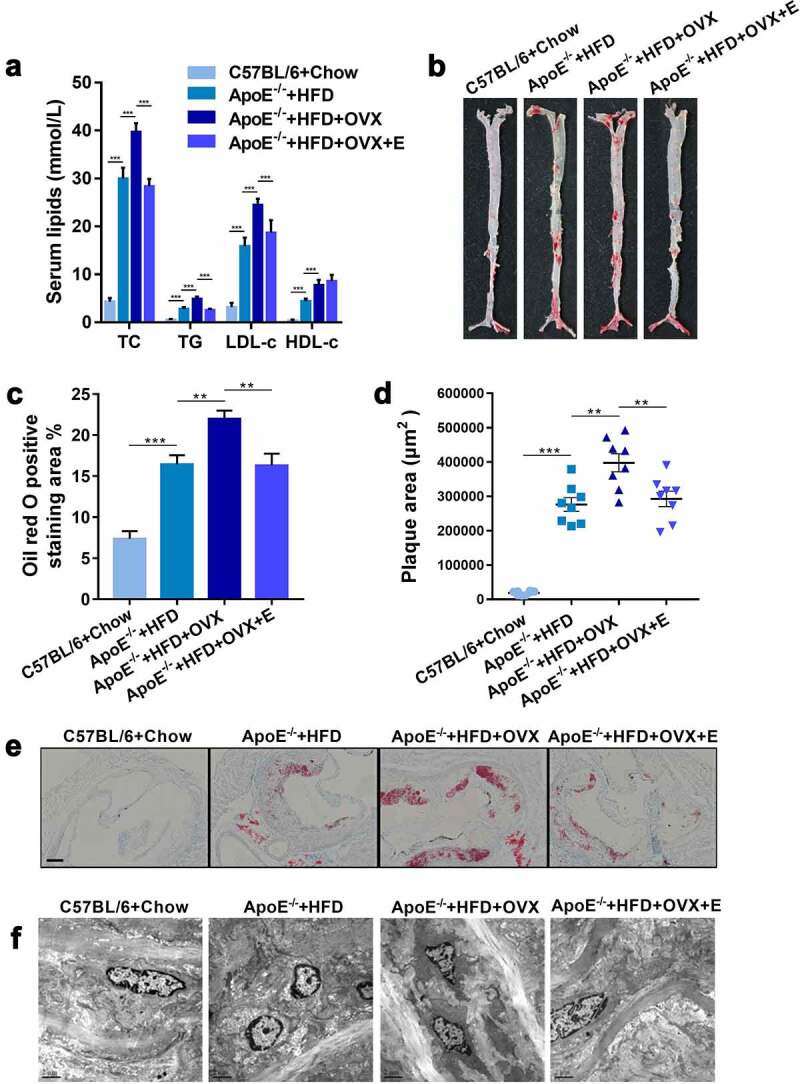
Plasma lipid levels and atherosclerotic lesions in mice in different groups

To evaluate the potential effects of estrogen on the progression of AS, the serum lipid levels (TC, triglycerides (TG), LDL-c and HDL-c) and atherosclerotic lesions in different groups during 90 days were detected. Compared with normally fed C57BL/6 mice, ApoE^−/-^ mice fed with a high-fat diet (HFD) had higher TC, TG, LDL-c, and HDL-c levels (Figure 1(a)). In addition, bilateral ovariectomy for HFD-fed ApoE^−/-^ mice significantly increased the levels of TC, TG, and LDL-c but decreased that of HDL-c compared to those of the mice without receiving surgery. Furthermore, HFD-fed ApoE^−/-^ mice showed significant aggravation of AS at the thoracic aorta (Figure 1(b,c)) and aortic root (Figure 1(d,e)) as well as significant increase of intima-media thickness (Figure 1(f)) compared with those of normally fed C57BL/6 mice. Bilateral ovariectomy also exacerbated atherosclerotic lesions in HFD-fed ApoE^−/-^ mice (Figure 1(b-f)). Collectively, estrogen deficiency accelerated the progression of AS, as reported previously **^[13]^**.

(a) Plasma TC, TG, LDL-c and HDL-c levels. The results are represented as mean ± SEM. n = 8. (b) Representative digital photographs of oil red O-stained thoracic aortas. (c) Percentages of lipid deposition areas in oil red O-stained thoracic aortas. n = 8. (d) Quantification of plaque areas in oil red O-stained thoracic aortic roots. n = 8. (e) Representative photomicrographs of oil red O-stained thoracic aortic roots. Scale bar = 100 μm.(f) Representative scanning electron microscopy micrographs of thoracic aorta cross-sections. n = 3.All values are provided in Supplementary Table 1 (for Figure 1(a,c,d)). **P < .01, ***p < .001. Non-significant comparisons are indicated by either “ns” or no asterisk.

After HFD-fed ApoE^−/-^ mice undergoing bilateral ovariectomy, they were intragastrical administered with 0.13 mg/kg estrogen for 90 days, the levels of TC, TG, and LDL-c were significantly reduced whereas HDL-c was significantly increased in them, when compared to those of HFD-fed ApoE^−/-^ mice without receiving surgery (Figure 1(a)). Furthermore, estrogen supplementation relieved the atherosclerotic lesions aggravated by HFD and ovariectomy, manifested as the reduction of lipid deposits in the thoracic aorta (Figure 1(b,c)) and aortic root (Figure 1(d,e)) together with intima-media damage (Figure 1(f)).

### 2.2. Estrogen supplementation reversed the lipid accumulation and the changes of mRNA expressions of lipid metabolism – related enzymes in ovariectomized HFD-fed ApoE^−/-^ mice

**Figure 2.**
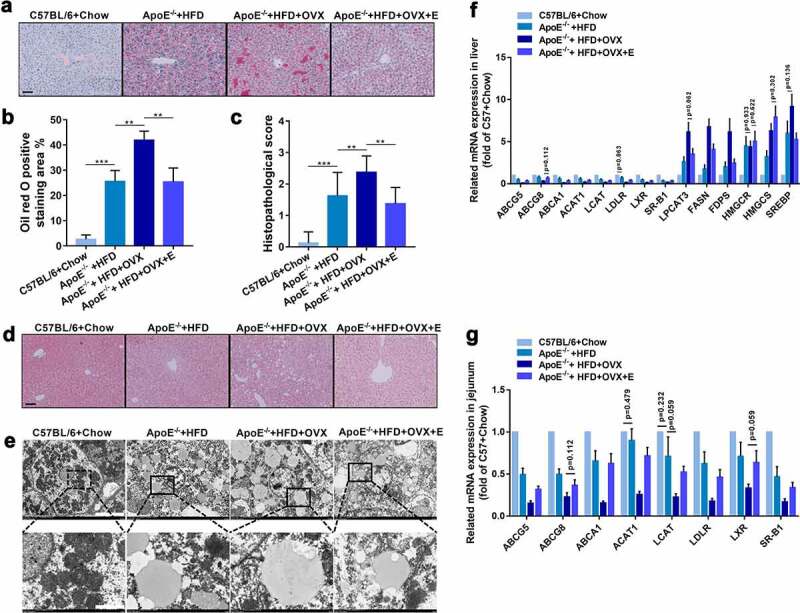
Hepatic histology and mRNA expressions of lipid metabolism – related enzymes in mice in different groups

Compared with HFD-fed ApoE^−/-^ mice without receiving ovariectomy, the loss of estrogen due to ovariectomy promoted lipid accumulation and tissue damage in the liver (Figure 2(a-e)). On the other hand, the mRNA levels of lipid metabolism-related enzymes in the liver, such as LPCAT3, FASN, FDPS, HMGCR HMGCS, and SREBP, significantly increased, but those of lipid transport enzymes, such as ABCG5, ABCG8, ABCA1, ACAT1, LCAT, LDLR, LXR, and SR-B1, significantly decreased. Supplementing ovariectomized HFD-fed ApoE^−/-^ mice with estrogen not only reduced lipid accumulation and tissue damage in the liver, but also significantly down-regulated the mRNA levels of lipid metabolism-related enzymes and up-regulated those of lipid transport enzymes (Figure 2(f)). We also studied the mRNA expressions of lipid transport enzymes in the intestine (jejunum). Ovariectomy decreased the expressions of ABCG5, ABCG8, ABCA1, ACAT1, LCAT, LDLR, LXR and SR-B1 in the small intestine (jejunum) of HFD-fed ApoE^−/-^ mice. Consistently, estrogen supplementation up-regulated the mRNA expressions of these lipid transport enzymes in the intestine (Figure 2(g)).

(a) Representative photomicrographs of oil red O-stained liver tissues. Scale bar = 50 μm.(b) Quantification of lipid deposition areas of oil red O-stained liver tissues. n = 8.(c) Histopathologic scores of HE-stained liver tissues. n = 8.(d) Representative photomicrographs of HE-stained liver tissues. Scale bar = 50 μm.(e) Representative scanning electron microscopy micrographs of liver sections. Scale bar = 10 μm in up panel. Scale bar = 2 μm in down panel. n = 3.(f) Expression levels of indicated genes in livers. The results are represented as mean ± SEM. n = 8. Except for indicated *p* values, all other differences between C57BL/6 + Chow group and ApoE^−/-^ + HFD group, between ApoE^−/-^ + HFD group and ApoE^−/-^ + HFD + OVX group, and between ApoE^−/-^ + HFD + OVX group and ApoE^−/-^ + HFD + OVX + E group had statistical significances (*p* < .05).(g) Expression levels of indicated genes in small intestines. The results are represented as mean ± SEM. n = 8. Except for indicated *p* values, all other differences between C57BL/6 + Chow group and ApoE^−/-^ + HFD group, between ApoE^−/-^ + HFD group and ApoE^−/-^ + HFD + OVX group, and between ApoE^−/-^ + HFD + OVX group and ApoE^−/-^ + HFD + OVX + E group had statistical significances (*p* < .05).All values are provided in Supplementary Table 2 (for Figure 2(b,c,f,g)). **P < .01, ***p < .001. Non-significant comparisons are indicated by either “ns” or no asterisk in B and C.

### 2.3. Lipid metabolomics were significantly changed in ovariectomized HFD-fed ApoE^−/-^ mice and reversed by estrogen supplementation

**Figure 3.**
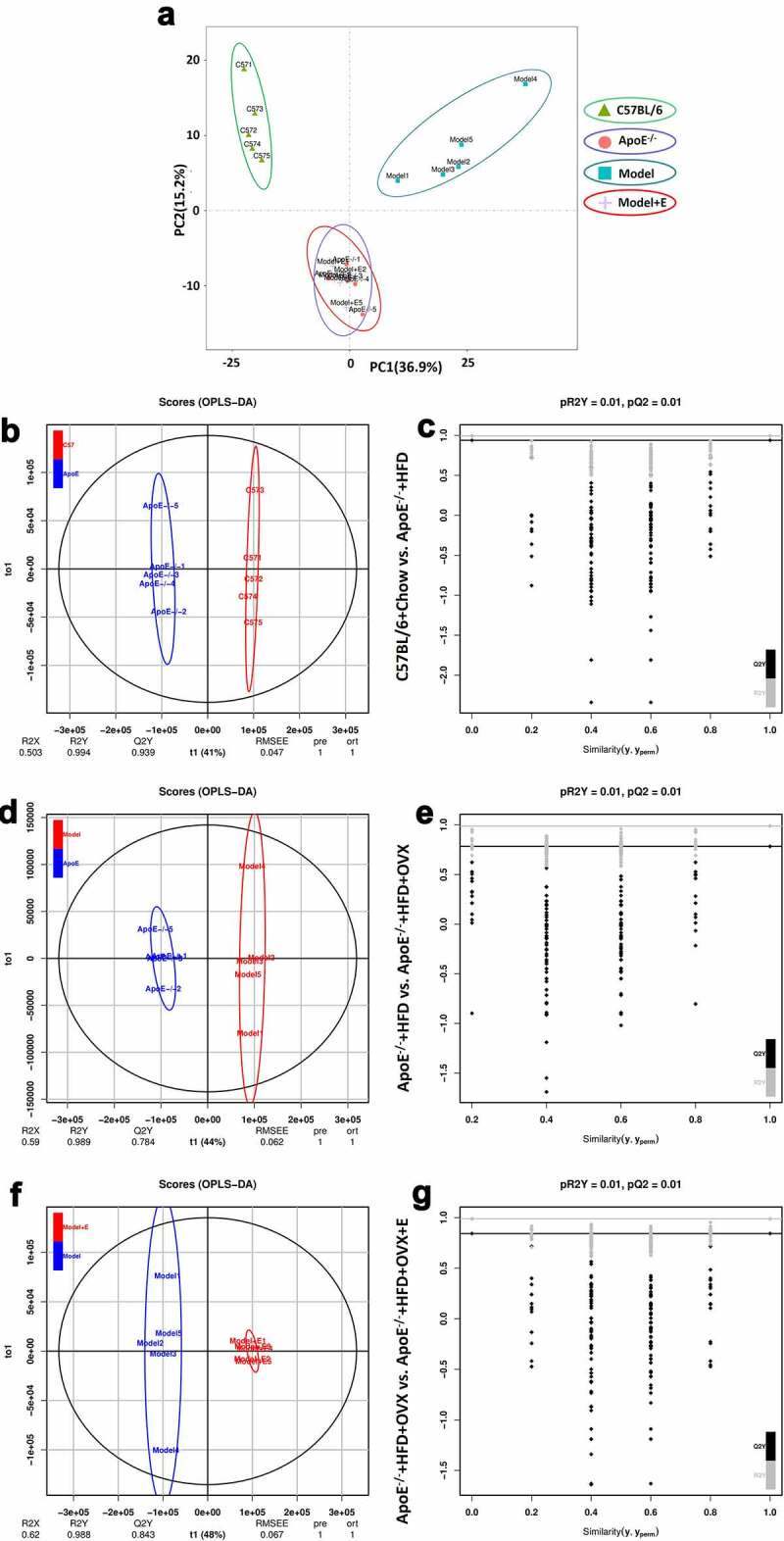
PCA, OPLS-DA, and Permutation test of serum lipid metabolites in mice in different groups

As an unsupervised pattern analytical method, principal component analysis (PCA) produces a new characteristic variable by a weighted linear combination of metabolites. The main data are classified by main new variables (principal components). PCA of serum samples revealed a clear separation of circulating lipid metabolites in normal diet-fed C57BL/6 mice, HFD-fed ApoE^−/-^ mice, ovariectomized HFD-fed ApoE^−/-^ mice, and estrogen-treated ovariectomized HFD-fed ApoE^−/-^ mice (Figure 3(a)), which revealed that lipid metabolomics was changed by modeling and bilateral ovariectomy-induced metabolic disturbances were regulated by estrogen supplementation. It was straightforward that estrogen treatment restored the effect of ovariectomy in HFD-fed ApoE^−/-^ mice.

(a) PCA plot of serum lipid metabolite profiling.(b) OPLS-DA plot of serum lipid metabolite profiling of C57BL/6 + Chow and ApoE^−/-^ + HFD groups.(c) Permutation test plot of serum lipid metabolite profiling of C57BL/6 + Chow and ApoE^−/-^ + HFD groups.(d) OPLS-DA plot of serum lipid metabolite profiling of ApoE^−/-^ + HFD and ApoE^−/-^ + HFD + OVX groups.(e) Permutation test plot of serum lipid metabolite profiling of ApoE^−/-^ + HFD and ApoE^−/-^ + HFD + OVX groups.(f) OPLS-DA plot of serum lipid metabolite profiling of ApoE^−/-^ + HFD + OVX and ApoE^−/-^ + HFD + OVX + E groups.(g) Permutation test plot of serum lipid metabolite profiling of ApoE^−/-^ + HFD + OVX and ApoE^−/-^ + HFD + OVX + E groups.C57BL/6 + Chow = C57BL/6, ApoE^−/-^ + HFD = ApoE^−/-^, ApoE^−/-^ + HFD + OVX = Model, ApoE^−/-^ + HFD + OVX + E = Model + E.All data are provided in Supplementary Table 3. n = 5.

Orthogonal partial least squares discriminant analysis (OPLS-DA) was also used to analyze metabolomics data, allowing the visualization and depiction of general metabolic variations between two groups. R2X, R2Y, and Q2Y represent the interpretability of independent variables, the interpretability of dependent variables and the predictability of OPLS-DA, respectively (Figure 3(b,d,f). The permutation test can be used to evaluate whether OPLS-DA is overfitting, with R2Y and Q2 representing the goodness-of-fit coefficients. No comparisons between every two groups showed overfitting (Figure 3(c,e,g)). The interpretabilities of OPLS-DA between normal diet-fed C57BL/6 mice and HFD-fed ApoE^−/-^ mice, between HFD-fed ApoE^−/-^ mice and ovariectomized HFD-fed ApoE^−/-^ mice, as well as between ovariectomized HFD-fed ApoE^−/-^ mice and estrogen-treated ovariectomized HFD-fed ApoE^−/-^ mice exceeded 0.5, and the discrimination between every two groups was larger. There were remarkable separations between the indicated two groups (Figure 3(b,d,f)).

**Figure 4.**
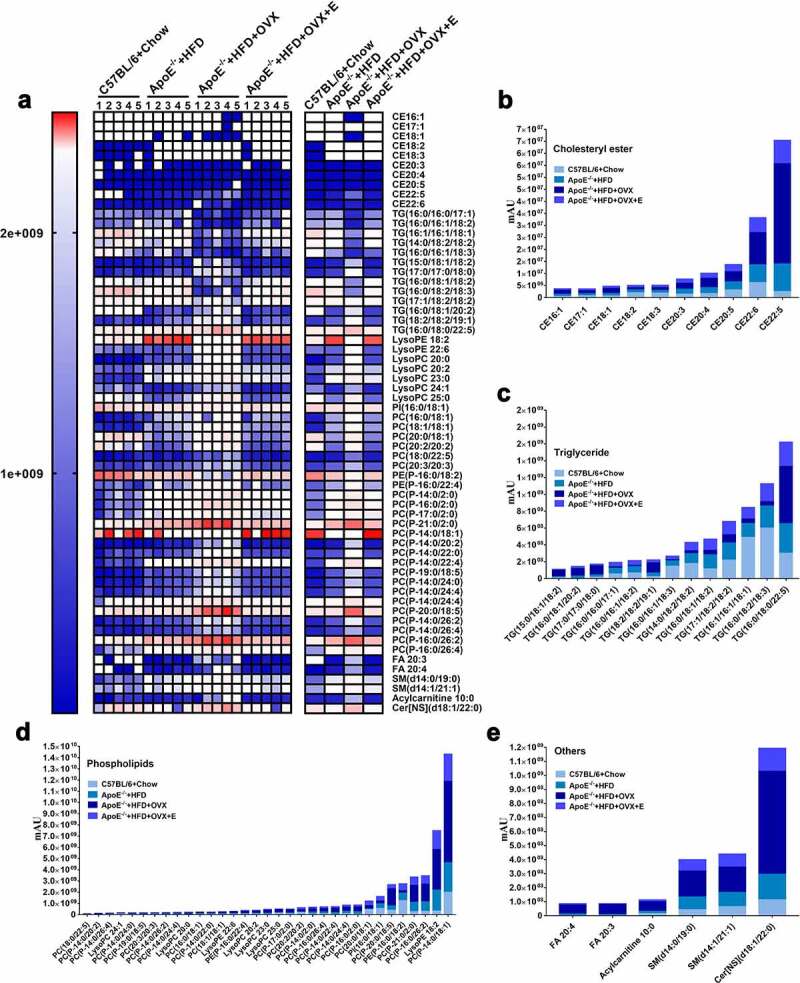
Heat map and cluster histogram of the serum lipid metabolites in mice in different groups

Then, we generated a heat map and cluster histograms from the lipid metabolomics to visualize and to depict the distinction between groups (Figure 4). Overall, the plasma levels of cholesterol esters (CE), TG, phospholipids, and other types of lipids (including free fatty acids, acylcarnitine, sphingomyelins, and ceramides) in ovariectomized HFD-fed ApoE^−/-^ mice were significantly higher than those of HFD-fed ApoE^−/-^ mice without receiving surgery (Figure 4). Particularly, compared with HFD-fed ApoE^−/-^ mice, the contents of nine kinds of CEs significantly increased whereas that of one kind (CE 18:2) significantly decreased in ovariectomized HFD-fed ApoE^−/-^ mice (Figure 4(a,b)). Estrogen administration (0.13 mg/kg, intragastrical) significantly reduced the contents of 9 kinds of CEs but increased that of CE 18:2 in the plasma of ovariectomized HFD-fed ApoE^−/-^ mice, making the levels approach those of HFD-fed ApoE^−/-^ mice. The contents of five kinds TGs significantly increased but those of eight kinds significantly decreased in ovariectomized HFD-fed ApoE^−/-^ mice, which were restored by estrogen administration to be close to those of HFD-fed ApoE^−/-^ mice (Figure 4(a,c)). The contents of 31 kinds of phospholipids significantly increased whereas those of 2 kinds significantly decreased in ovariectomized HFD-fed ApoE^−/-^ mice, which were recovered by estrogen administration to approach those of HFD-fed ApoE^−/-^ mice (Figure 4(a,d)). The contents of two kinds of free fatty acids, two kinds of sphingomyelins, one kind of acylcarnitine and one kind of ceramide significantly increased in ovariectomized HFD-fed ApoE^−/-^ mice, which were significantly reduced by estrogen administration to be close to those of HFD-fed ApoE^−/-^ mice (Figure 4(a,e)).

(a) Heat map of serum lipid metabolites, with colors indicating relative abundances. Blue means lowest, and red means highest. Except for comparisons between ApoE^−/-^ + HFD + OVX group and ApoE^−/-^ + HFD + OVX + E group concerning CE 18:2, CE 18:3, CE 20:3, CE 20:5 and CE 22:6, all other differences between ApoE^−/-^ + HFD group and ApoE^−/-^ + HFD + OVX group, and between ApoE^−/-^ + HFD + OVX group and ApoE^−/-^ + HFD + OVX + E group had statistical significances (*p* < .05).(b) Related to Figure 4(a), cluster histogram of serum cholesterol ester metabolites.(c) Related to Figure 4(a), cluster histogram of serum triglyceride metabolites.(d) Related to Figure 4(a), cluster histogram of serum phospholipid metabolites.(e) Related to Figure 4(a), cluster histogram of other types of serum lipid metabolites (including free fatty acids, acylcarnitine, sphingomyelins and ceramides).All data are provided in Supplementary Table 3. n = 5.

### 2.4. Estrogen supplementation called back the changing of the gut microbiota caused by ovariectomy in HFD-fed ApoE^−/-^ mice

**Figure 5.**
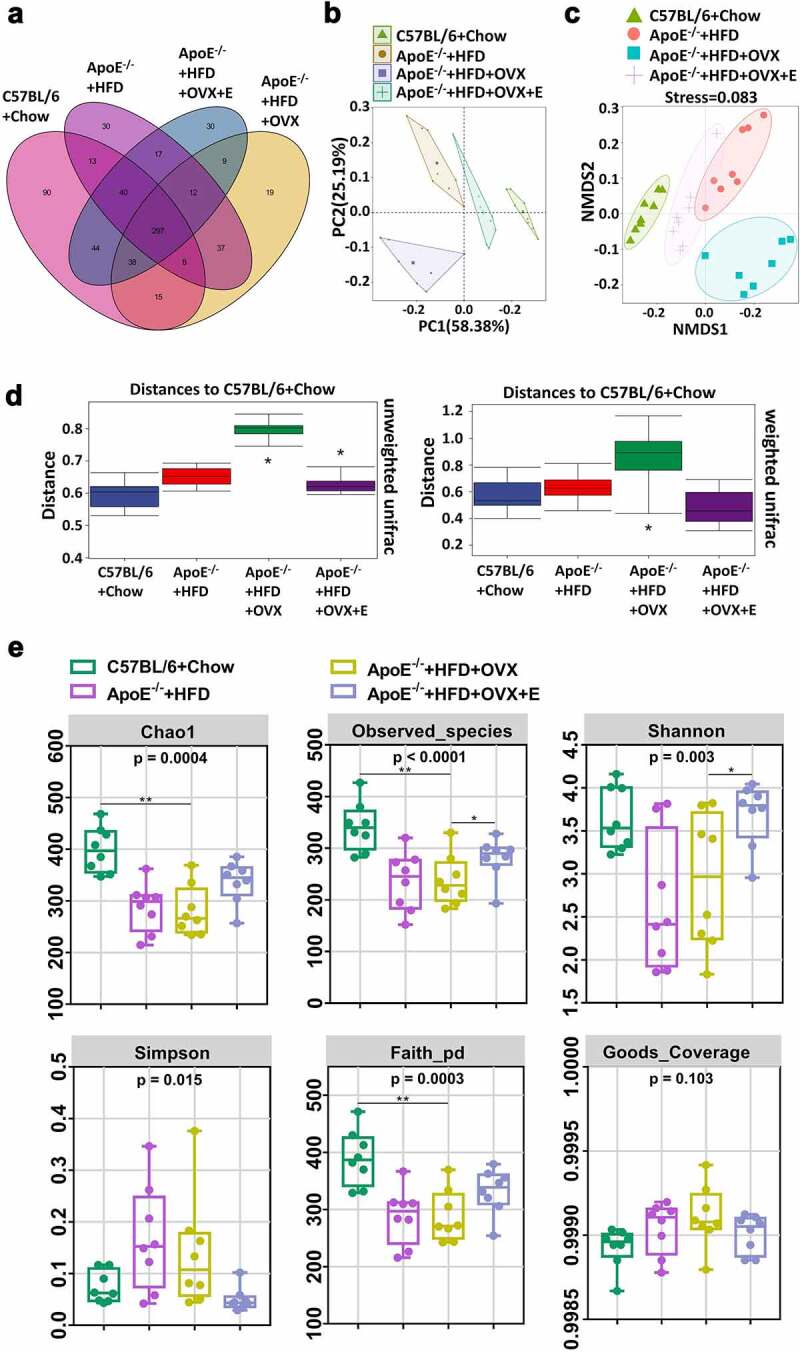
Cluster and diversity analysis of gut microbiota in mice in different groups

Based on the operational taxonomic unit (OTU) abundance table, the Venn graph gives the unique OTUs in each group and the shared OTUs between groups. There were 545 OTUs and 90 unique OTUs in normal diet-fed C57BL/6 mice, 454 OTUs and 23 unique OTUs in HFD-fed ApoE^−/-^ mice, 435 OTUs and 16 unique OTUs in ovariectomized HFD-fed ApoE^−/-^ mice, and 487 OTUs and 30 unique OTUs in estrogen-treated ovariectomized HFD-fed ApoE^−/-^ mice. Normal diet-fed C57BL/6 mice had the most gut microbiota species (OTUs), followed by the HFD-fed ApoE^−/-^ mice, ovariectomized HFD-fed ApoE^−/-^ mice and estrogen-treated ovariectomized HFD-fed ApoE^−/-^ mice sequentially (Figure 5(a)). PCA showed that there was a clear separation of gut microbiota species in the fecal samples of the four groups. There were significant differences in the gut microbiota species between normal diet-fed C57BL/6 mice and HFD-fed ApoE^−/-^ mice, between HFD-fed ApoE^−/-^ mice and ovariectomized HFD-fed ApoE^−/-^ mice, as well as between ovariectomized HFD-fed ApoE^−/-^ mice and estrogen-treated ovariectomized HFD-fed ApoE^−/-^ mice. The gut microbiota species in ovariectomized HFD-fed ApoE^−/-^ mice were recovered by estrogen supplementation to be close to those in HFD-fed ApoE^−/-^ mice and even to normal diet-fed C57BL/6 mice (Figure 5(b)). Six kinds of analysis (Chao1 and Observed species are used to characterize richness, Shannon and Simpson) are used to characterize diversity, Faith PD is used to characterize evolution-based diversity, and Good’s coverage is used to characterize coverage) were used to display the α diversity in gut microbiota in mice feces. The richness, diversity, and evolution-based diversity in HFD-fed ApoE^−/-^ mice were significant decreased by ovariectomy. Estrogen supplementation increased richness, diversity, and evolution-based diversity of gut microbiota caused by ovariectomy in HFD-fed ApoE^−/-^ mice (Figure 5(e)). The same changes in the four groups were present in β diversity of gut microbiota, which were analyzed by NMDS and difference analysis (Figure 5(c,d)).

(a) Venn diagram of the gut microbiota in feces. (b) PCA plot of the gut microbiota in feces. (c) Beta diversity analysis of NMDS plot of the gut microbiota in feces. (d) Beta diversity analysis of difference analysis of the gut microbiota in feces. Distance algorithm of unweighted unifrac was used in left panel. Distance algorithm of weighted unifrac was used in right panel. (e) Alpha diversity analysis of the gut microbiota in feces. All data are provided in Supplementary Table 4. *P < .05, **p < .01. Non-significant comparisons are indicated by either “ns” or no asterisk in D.

**Figure 6.**
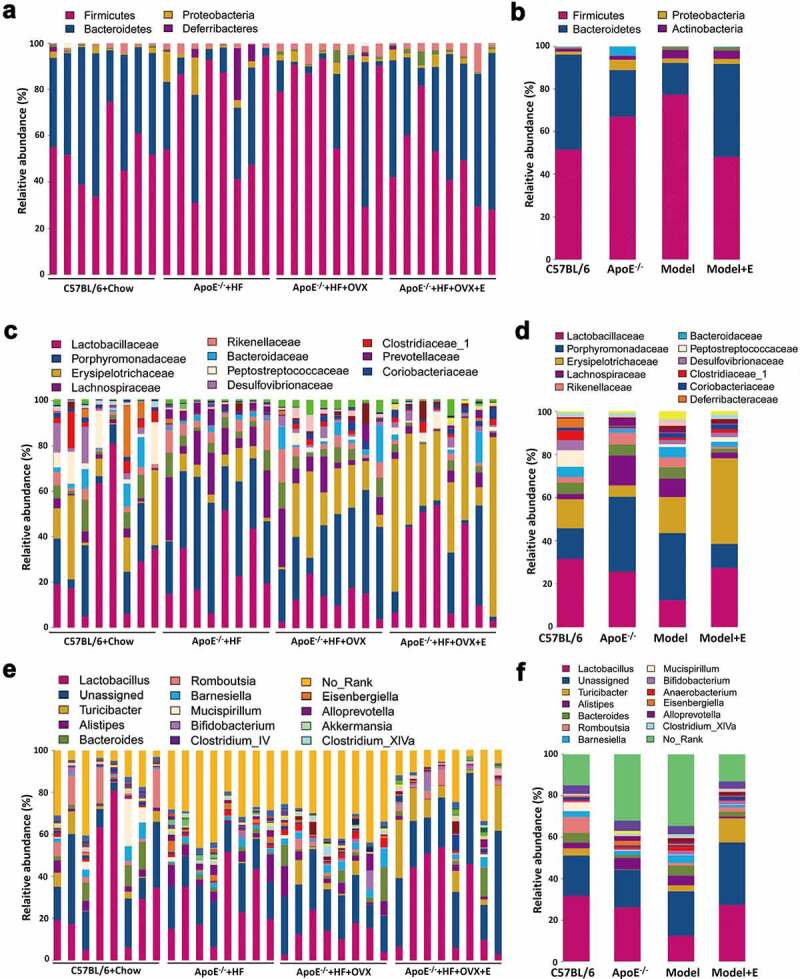
Relative abundance analysis of gut microbiota in mice in different groups

We then plotted cluster histograms to show the compositions of gut microbiota species in each sample (Figures 6(a,c,e)) and in each group (Figures 6(b,d,f)). At the phylum level, the gut microbiotas in the fecal samples of the four groups were dominated by *Bacteroidetes* and *Firmicutes*. The gut microbiota composition of HFD-fed ApoE^−/-^ mice was significantly different from that of normal diet-fed C57BL/6 mice. The relative abundances of *Firmicutes* and *Proteobacteria* increased, whereas that of *Bacteroidetes* decreased. Compared to HFD-fed ApoE^−/-^ mice, the above-mentioned changes were further enhanced in ovariectomized HFD-fed ApoE^−/-^ mice. The relative abundances of *Firmicutes, Bacteroidetes* and *Proteobacteria* in estrogen-treated ovariectomized HFD-fed ApoE^−/-^ mice were like those of normal diet-fed C57BL/6 mice (Figures 6(a,b)). At the family level, the relative abundances of *Lactobacillaceae, Clostridiaceae, Desulfovibrionaceae* and *Peptostreptococcaceae* in the feces of normal diet-fed C57BL/6 mice were significantly higher than those of other groups. The relative abundances of *Porphyromonadaceae, Lachnospiraceae, Rikenellaceae* and *Prevotellaceae* in the gut microbiota of HFD-fed ApoE^−/-^ mice significantly surpassed those of normal diet-fed C57BL/6 mice. The ovariectomized HFD-fed ApoE^−/-^ mice had significantly higher family levels of *Erysipelotrichaceae, Bacteroidaceae* and *Coriobacteriaceae* in the gut microbiota than those of other groups. Compared to ovariectomized HFD-fed ApoE^−/-^ mice, the relative abundances of *Lactobacillaceae* and *Erysipelotrichaceae* increased, but those of *Porphyromonadaceae and Lachnospiraceae* decreased after estrogen supplementation (Figures 6(c,d)). At the genus level, the relative abundances of *Akkermansia, Clostridium_XlVa* and *Lactococcus* in the gut microbiota of the feces from normal diet-fed C57BL/6 mice were significantly higher than those of other groups. The relative abundances of *Ruminococcus, Escherichia/Shigella* and *Alloprevotella* in the gut microbiota of HFD-fed ApoE^−/-^ mice significantly exceeded those of normal diet-fed C57BL/6 mice. The ovariectomized HFD-fed ApoE^−/-^ mice had significantly higher levels of *Turicibacter, Streptococcus, Prevotella* and *Enterorhabdus* than those of other groups. Compared to ovariectomized HFD-fed ApoE^−/-^ mice, the relative abundances of *Enterococcus* and *Lactobacillus* increased, whereas that of *Prevotella* decreased after estrogen supplementation (Figure 6(e,f)).

(a) Relative abundances of the gut microbiota in feces at the phylum level. Each column corresponds to one sample. n = 8. (b) Related to Figure 6(a), also showing the relative abundances in feces at the phylum level. Each column corresponds to the mean of total samples in each group. (c) Relative abundances of the gut microbiota in feces at the family level. Each column corresponds to one sample. n = 8. (d) Related to Figure 6(b), also showing the relative abundances in feces at the family level. Each column corresponds to the mean of total samples in each group. (e) Relative abundances of the gut microbiota in feces at the genus level. Each column corresponds to one sample. n = 8. (f) Related to Figure 6(c), also showing the relative abundances in feces at the genus level. Each column corresponds to the mean of total samples in each group. In 6B,6D, and 6 F, C57BL/6 + Chow = C57BL/6, ApoE^−/-^ + HFD = ApoE^−/-^, ApoE^−/-^ + HFD + OVX = Model, ApoE^−/-^ + HFD + OVX + E = Model + E.All data are provided in Supplementary Table 4.

### 2.5. Fecal microbiota transplantation remodeled gut microbiota and alleviated atherosclerosis in ovariectomized HFD-fed ApoE^−/-^ mice

**Figure 7.**
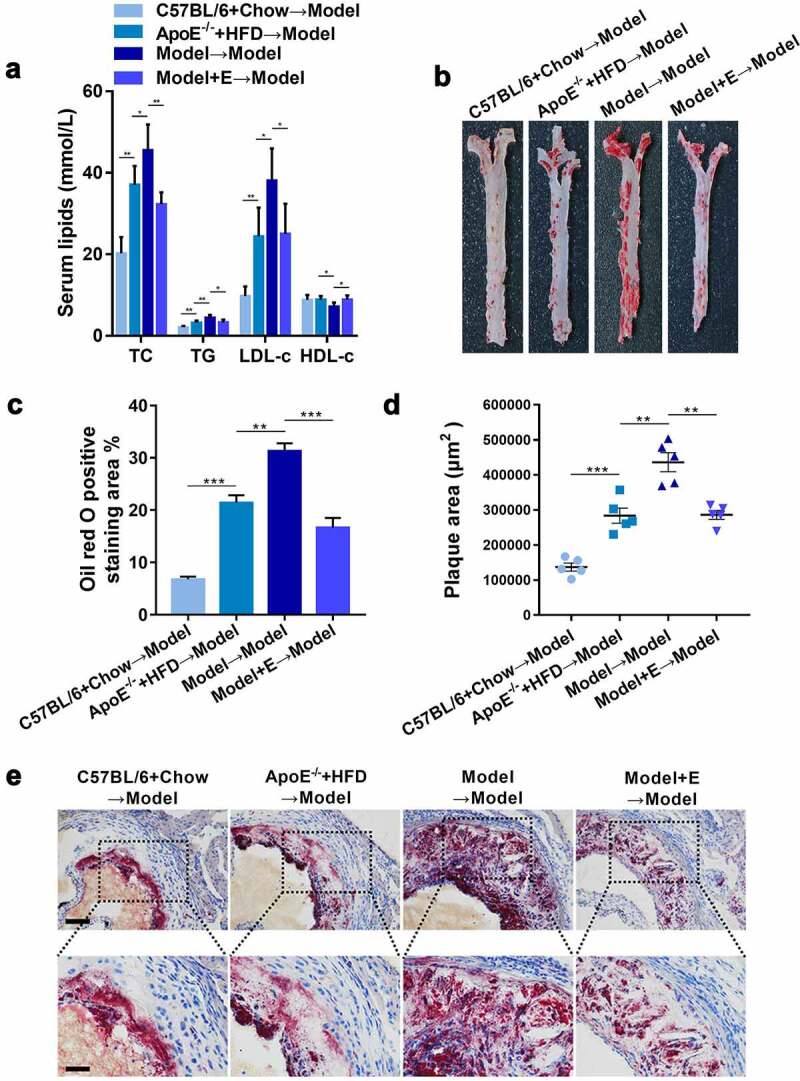
Plasma lipid levels and atherosclerotic lesions in ovariectomized HFD-fed ApoE^−/-^ mice with fecal microbiota transplantation

We then explored whether the fecal microbiota transplantation to ovariectomized HFD-fed ApoE^−/-^ mice can improve the atherosclerosis injury. The levels of TC, TG, and LDL-c were significantly reduced whereas HDL-c was significantly increased in ovariectomized HFD-fed ApoE^−/-^ mice accepted fecal microbiota transplantation from estrogen-treated ovariectomized HFD-fed ApoE^−/-^ mice (Figure 7(a)). Furthermore, the atherosclerotic lesions aggravated by ovariectomy in HFD-fed ApoE^−/-^ mice were decreased by fecal microbiota transplantation from estrogen-treated ovariectomized HFD-fed ApoE^−/-^ mice, including the reduction of lipid deposits in the thoracic aorta (Figure 7(b,c)) and aortic root (Figure 7(d,e)). All these beneficial changes could also be observed in ovariectomized HFD-fed ApoE^−/-^ mice accepted fecal microbiota transplantation from HFD-fed ApoE^−/-^ mice or normal diet-fed C57BL/6 mice (Figure 7).

(a) Plasma TC, TG, LDL-c and HDL-c levels. The results are represented as mean ± SEM. n = 5.(b) Representative digital photographs of oil red O-stained thoracic aortas.(c) Percentages of lipid deposition areas in oil red O-stained thoracic aortas. n = 5.(d) Quantification of plaque areas in oil red O-stained thoracic aortic roots. n = 5.(e) Representative photomicrographs of oil red O-stained thoracic aortic roots. Scale bar = 100 μm in up panel. Scale bar = 200 μm in down panel. Model = ApoE^−/-^ + HFD + OVX + E. All values are provided in Supplementary Table 1 (for Figure 7A, 7 C, and 7D). *P < .05, **p < .01, ***p < .001. Non-significant comparisons are indicated by either “ns” or no asterisk.

**Figure 8.**
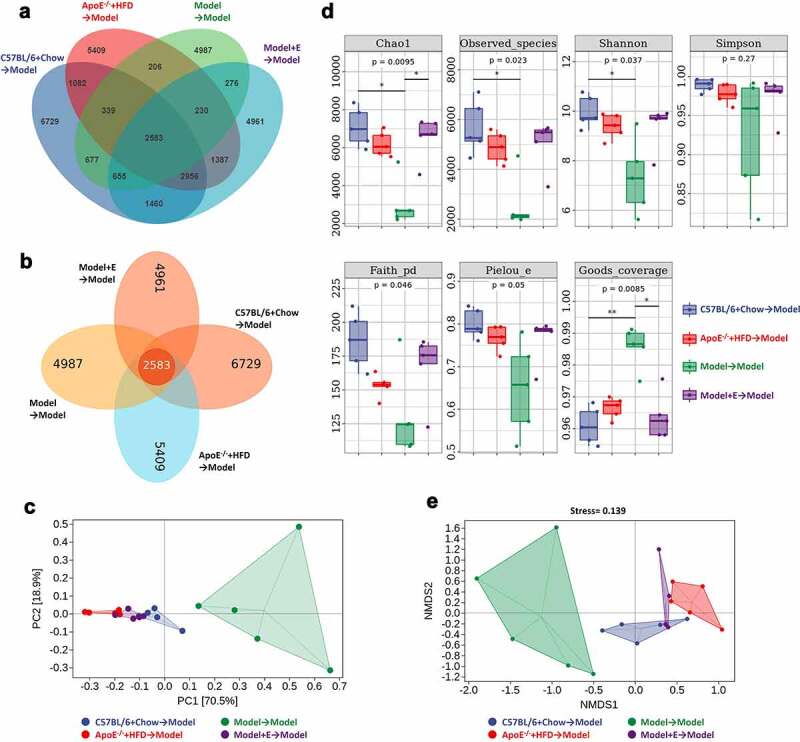
Cluster and diversity analysis of gut microbiota in ovariectomized HFD-fed ApoE^−/-^ mice with fecal microbiota transplantation

Consistent with our speculation, fecal microbiota transplantation changed the composition of the gut microbiota of ovariectomized HFD-fed ApoE^−/-^ mice. The typical and simple Venn graphs gave the unique OTUs in each group and the shared OTUs between groups. The gut microbiota species (OTUs) in ovariectomized HFD-fed ApoE^−/-^ mice accepted fecal microbiota transplantation from C57BL/6 group had the highest abundance, followed by the mice accepted fecal microbiota transplantation from HFD-fed ApoE^−/-^ mice, ovariectomized HFD-fed ApoE^−/-^ mice, and estrogen-treated ovariectomized HFD-fed ApoE^−/-^ mice (Figure 8(a,b)). PCA showed that there was a clear separation of gut microbiota species in the fecal samples of ovariectomized HFD-fed ApoE^−/-^ mice accepted fecal microbiota transplantation from the same treated mice from other three groups (Figure 8(c)). Seven kinds analysis were used to display the α diversity in gut microbiota in mice feces, ovariectomized HFD-fed ApoE^−/-^ mice accepted fecal microbiota transplantation from the same treated mice showed the lowest richness and diversity in gut microbiota in all groups (Figure 8(d)). NMDS was performed to display the β diversity in gut microbiota in mice feces, it was obvious that the β diversity in gut microbiota in ovariectomized HFD-fed ApoE^−/-^ mice accepted fecal microbiota transplantation from the same treated mice showed significant separation from other three groups (Figure 8(e)).

(a) Typical Venn diagram of the gut microbiota in feces. (b) Simple Venn diagram (displays independent and co-owned Out) of the gut microbiota in feces. (c) PCA plot of the gut microbiota in feces. (d) Alpha diversity analysis of the gut microbiota in feces. (e) Beta diversity analysis of NMDS plot of the gut microbiota in feces. Model = ApoE^−/-^ + HFD + OVX + E. All data are provided in Supplementary Table 5. *P < .05, **p < .01. Non-significant comparisons are indicated by either “ns” or no asterisk in C.

**Figure 9.**
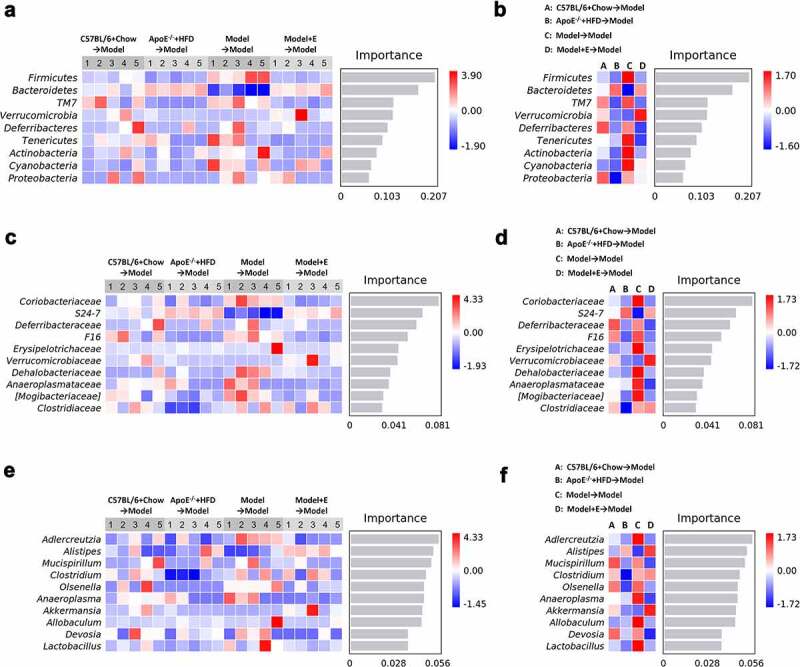
Relative abundance analysis of gut microbiota in ovariectomized HFD-fed ApoE^−/-^ mice with fecal microbiota transplantation

Random forest classifier was used to show the changing of compositions of gut microbiota species in each sample (Figure 9(a,c,e)) and in each group (Figure 9(b,d,f)) at the phylum level (Figure 9(a,b)), family level (Figure 9(c,d)), and genus level (Figure 9(e,f)). Random forest classifier counted the 10 categories with the most significant differences between groups at different levels (only 9 categories at the phylum level have significant differences), and ranked them in order of importance. At the phylum level, the changing of *Bacteroidetes* and *Firmicutes* in each group was like that in the feces of donor mice, that is, ovariectomized HFD-fed ApoE^−/-^ mice accepted fecal microbiota transplantation from the same treated mice had the highest relative abundances of *Firmicutes* and lowest *Bacteroidetes* (Figure 6(a,b), 9(a,b)). After ovariectomized HFD-fed ApoE^−/-^ mice accepted fecal microbiota transplantation from normal diet-fed C57BL/6 mice, HFD-fed ApoE^−/-^ mice, or estrogen-treated ovariectomized HFD-fed ApoE^−/-^ mice, the relative abundances of *Firmicutes* was decreased and *Bacteroidetes Firmicutes* was increased (Figure 9(a,b)). At the family level, the changing of *Desulfovibrionaceae* in each group was like that in the feces of donor mice. But the relative abundances of *Clostridiaceae* in each group showed completely opposite changes in the feces of recipient and donor mice (Figure 6(c,d), 9(c,d)). The relative abundance of *Clostridiaceae* was decreased and *Desulfovibrionaceae* was increased in ovariectomized HFD-fed ApoE^−/-^ mice accepted fecal microbiota transplantation from estrogen-treated ovariectomized HFD-fed ApoE^−/-^ mice compared to ovariectomized HFD-fed ApoE^−/-^ mice accepted fecal microbiota transplantation from ovariectomized HFD-fed ApoE^−/-^ mice (Figure 9(c,d)). At the genus level, the changing of *Akkermansia* in each group was like that in the feces of donor mice. But the relative abundances of *Lactococcus* in each group showed completely opposite changes in the feces of recipient and donor mice (Figures 6(e,f), 9(e,f)). The most changing species at the genus level in recipient mice were *Adlercreutzia* and *Alistipes*. The relative abundance of *Adlercreutzia* was decreased and *Alistipes* was increased in ovariectomized HFD-fed ApoE^−/-^ mice accepted fecal microbiota transplantation from estrogen-treated ovariectomized HFD-fed ApoE^−/-^ mice compared to ovariectomized HFD-fed ApoE^−/-^ mice accepted fecal microbiota transplantation from ovariectomized HFD-fed ApoE^−/-^ mice (Figure 9(e,f)).

(a) Relative abundances of the gut microbiota in feces at the phylum level. Each column corresponds to one sample. n = 5. (b) Related to Figure 9A, also showing the relative abundances in feces at the phylum level. Each column corresponds to the mean of total samples in each group. (c) Relative abundances of the gut microbiota in feces at the family level. Each column corresponds to one sample. n = 5. (d) Related to Figure 9B, also showing the relative abundances in feces at the family level. Each column corresponds to the mean of total samples in each group. (e) Relative abundances of the gut microbiota in feces at the genus level. Each column corresponds to one sample. n = 5. (f) Related to Figure 9C, also showing the relative abundances in feces at the genus level. Each column corresponds to the mean of total samples in each group. Model = ApoE^−/-^ + HFD + OVX + E. All data are provided in Supplementary Table 5.

### 2.6. Fecal microbiota transplantation reversed lipid metabolomics in ovariectomized HFD-fed ApoE^−/-^ mice

**Figure10.**
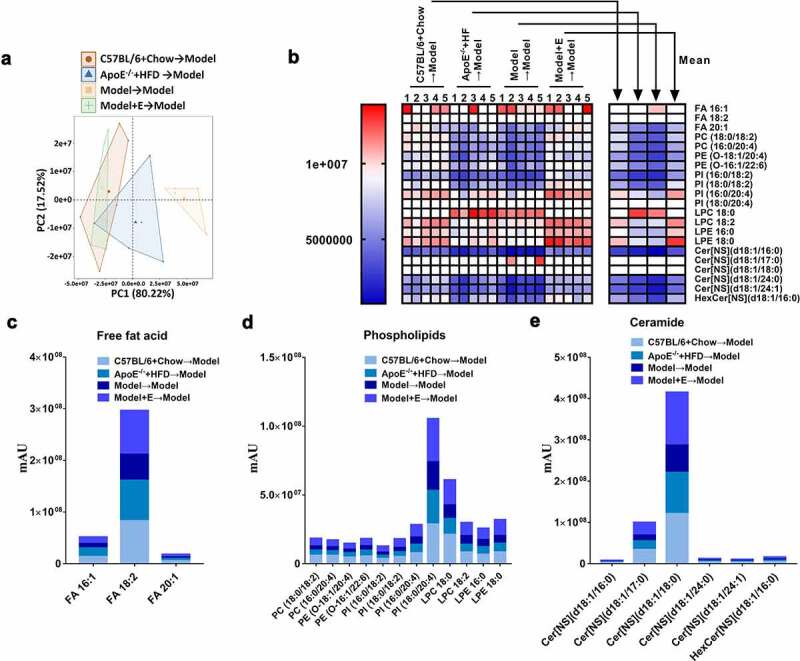
Changes of the serum lipid metabolites in ApoE^−/-^ mice with fecal microbiota transplantation

PCA of serum samples revealed a clear separation of circulating lipid metabolites in ovariectomized HFD-fed ApoE^−/-^ mice accepted fecal microbiota transplantation from the same treated mice from other three groups (Figure 10(a)), which revealed that lipid metabolomics was changed by adjusting gut microbiota through fecal microbiota transplantation, and revealed that estrogen treatment could reverse lipid metabolomics by changing the gut microbiota composition. The heat map and cluster histograms from the lipid metabolomics visualized and depicted the distinction in circulating lipid metabolites in fecal microbiota transplantation accepted mice (Figure 10(b)). Different from the donor mice, the number of changing species of lipid metabolomics in recipient mice was less, and the significantly varied species of lipid metabolomics in recipient mice mainly in free fatty acids, phospholipids, and ceramides (Figures 4(a), 10(b)). Specifically, compared to ovariectomized HFD-fed ApoE^−/-^ mice accepted fecal microbiota transplantation from the same treated mice, ovariectomized HFD-fed ApoE^−/-^ mice accepted fecal microbiota transplantation from normal diet-fed C57BL/6 mice, HFD-fed ApoE^−/-^ mice, or estrogen-treated ovariectomized HFD-fed ApoE^−/-^ mice, had two kids of free fat acid (FA16:1 and FA 18:1) decreased and one kind of fat acid (FA20:1) increased (Figure 10(b,c)). As for phospholipids, except for LPC 18:0, other 11 kinds of significantly varied phospholipids were higher in ovariectomized HFD-fed ApoE^−/-^ mice accepted fecal microbiota transplantation from estrogen-treated ovariectomized HFD-fed ApoE^−/-^ mice, compared to ovariectomized HFD-fed ApoE^−/-^ mice accepted fecal microbiota transplantation from ovariectomized HFD-fed ApoE^−/-^ mice (Figure 10(b,d)). The significantly varied species of ceramides (six kinds) in ovariectomized HFD-fed ApoE^−/-^ mice accepted fecal microbiota transplantation from the same treated mice were all the lowest compared to other three groups (Figure 10(b,e)).

(a) PCA plot of serum lipid metabolite profiling. (b) Heat map of serum lipid metabolites, with colors indicating relative abundances. Blue means lowest, and red means highest. All comparisons between Model → Model group and Model + E → Model group had statistical significances (*p* < .05).(d) Related to Figure 10 C, cluster histogram of serum-free fat acid metabolites.(e) Related to Figure 10 C, cluster histogram of serum phospholipid metabolites.(f) Related to Figure 10 C, cluster histogram of serum ceramide metabolites. Model = ApoE^−/-^ + HFD + OVX + E. All data are provided in Supplementary Table 6. n = 5.

### 2.7. The changing of gut microbiota and lipid metabolites showed significant correlation in mice

**Figure 11.**
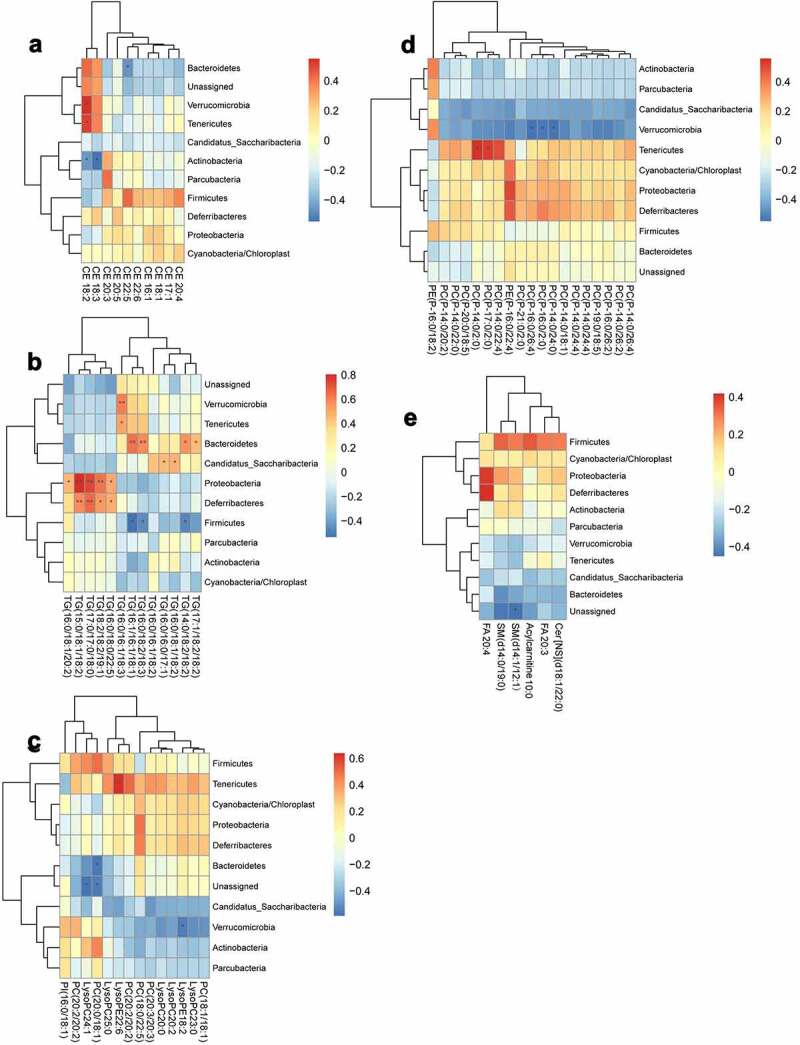
Analysis of the correlation between the lipid metabolites and gut microbiota at phylum level

**Figure 12.**
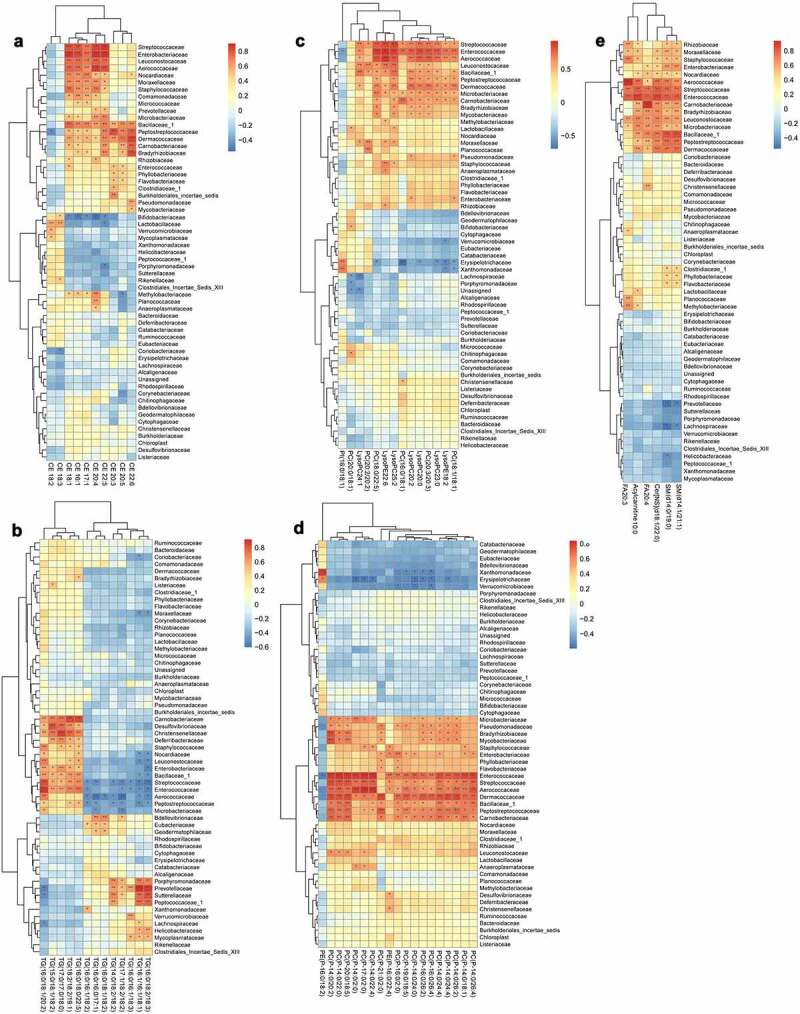
Analysis of the correlation between the lipid metabolites and gut microbiota at family level

**Figure 13.**
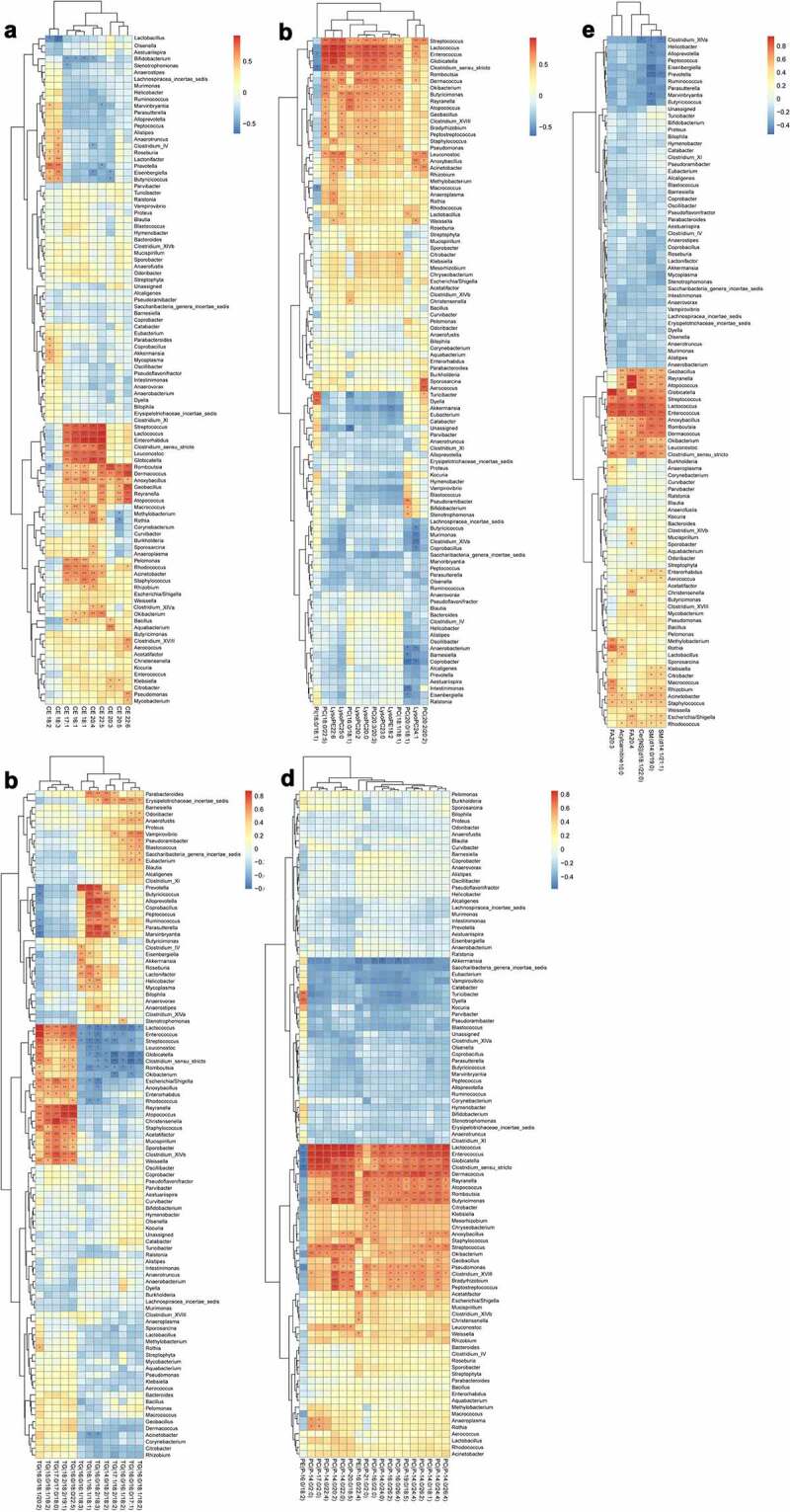
Analysis of the correlation between the lipid metabolites and gut microbiota at genus level

For correlation analysis, a total of 10 kinds of CE, 13 kinds of TG, 33 kinds of phospholipids and 6 other types of lipids (including free fatty acids, acylcarnitine, sphingomyelins, and ceramides) were included. Their correlations with the gut microbiota at the phylum level (Figure 11), family level (Figure 12) or genus level (Figure 13) were analyzed.

(a) Heat map of correlations between 10 kinds of cholesterol esters (CE) and the gut microbiota at the phylum level. (b) Heat map of correlations between 13 kinds of triglycerides (TG) and the gut microbiota at the phylum level. (c) Heat map of correlations between 14 kinds of phospholipids and the gut microbiota at the phylum level. (d) Heat map of correlations between 19 kinds of phospholipids and the gut microbiota at the phylum level. (e) Heat map of correlations between six other types of lipids and the gut microbiota at the phylum level.*P < .05, **p < .01. Non-significant comparisons are indicated by either “ns” or no asterisk. Red means positive correlation, and blue means negative correlation. The correlation analysis was performed without using the data from the mice participating in FMT experiment.

(a) Heat map of correlations between 10 kinds of cholesterol esters (CE) and the gut microbiota at the family level. (b) Heat map of correlations between 13 kinds of triglycerides (TG) and the gut microbiota at the family level. (c) Heat map of correlations between 14 kinds of phospholipids and the gut microbiota at the family level. (d) Heat map of correlations between 19 kinds of phospholipids and the gut microbiota at the family level. (e) Heat map of correlations between six other types of lipids and the gut microbiota at the family level.*P < .05, **p < .01. Non-significant comparisons are indicated by either “ns” or no asterisk. Red means positive correlation, and blue means negative correlation. The correlation analysis was performed without using the data from the mice participating in FMT experiment.

(a) Heat map of correlations between 10 kinds of cholesterol esters (CE) and the gut microbiota at the genus level. (b) Heat map of correlations between 13 kinds of triglycerides (TG) and the gut microbiota at the genus level. (c) Heat map of correlations between 14 kinds of phospholipids and the gut microbiota at the genus level. (d) Heat map of correlations between 19 kinds of phospholipids and the gut microbiota at the genus level. (e) Heat map of correlations between six other types of lipids and the gut microbiota at the genus level.*P < .05, **p < .01. Non-significant comparisons are indicated by either “ns” or no asterisk. Red means positive correlation, and blue means negative correlation. The correlation analysis was performed without using the data from the mice participating in FMT experiment.

At the phylum level, CE18:2 in CE was significantly positively correlated with *Verrucomicrobia* and *Tenericutes*, but negatively correlated with *Actinobacteria*, the same as CE18:3. CE22:5 was significantly negatively correlated with *Bacteroidetes*. A variety of TG showed positive correlations with *Bacteroidetes, Proteobacteria* and *Deferribacteres*. PC (*P*-14:0/2:0) and PC (*P*-17:0/2:0) in phospholipids showed significant positive correlations with *Tenericutes*. PC (*P*-16:0/26:4), PC (*P*-16:0/2:0) and PC (*P*-14:0/24:0) were negatively correlated with *Verrucomicrobia* (Figure 11).

At the family level, most kinds of CE were significantly positively correlated with *Streptococcaceae, Enterobacteriaceae, Leuconostocaceae, Aerococaceae, Nocardiaceae, Moraxellaceae, Staphylococcaceae, Bacillaceae, Peptostreptococcaceae, Dermacoccaceae, Carnobacteriaceae* and *Bradyrhizobiaceae*, whereas negatively correlated with *Bifidobacteriaceae*. Some TG were positively correlated with *Enterobacteriaceae* and negatively correlated with *Enterococcaceae*, but the remaining showed negative correlation. Almost all kinds of phospholipids were significantly positively correlated with *Streptococcaceae, Enterococcaceae, Aerococaceae, Leuconostocaceae, Bacillaceae, Peptostreptococcaceae, Dermacoccaceae, Microbacteriaceae, Carnobacteriaceae, Pseudomonadaceae, Bradyrhizobiaceae* and *Mycobacteriaceae*, and negatively correlated with *Erysipelotrichaceae. The* other types of lipids (including free fatty acids, acylcarnitine, sphingomyelins and ceramides) showed significant positive correlations with *Rhizobiaceae, Moraxellaceae, Staphylococcaceae, Enterobacteriaceae, Nocardiaceae, Aerococaceae, Streptococcaceae, Enterococcaceae, Carnobacteriaceae, Bradyrhizobiaceae, Leuconostocaceae, Microbacteriaceae, Bacillaceae, Peptostreptococcaceae* and *Dermacoccaceae* (Figure 12).

In comparison, the correlations between the gut microbiota at the genus level and lipid metabolomics were more complicated and detailed than those at the family level owing to existence of more species (Figure 13).

## 3. Discussion

The significant increase of CVD risk during menopause has been confirmed by many studies.^22–24^ As a risk factor for the progression of CVD,^25,26^ AS progression is significantly accelerated during menopause.^27,28^ In this study, bilateral ovariectomy promoted the progression of AS in HFD-fed ApoE^−/-^ mice, and estrogen supplementation inhibited AS lesion formation, revealing a potential link of estrogen to AS and CVD risk events.

Hypercholesterolemia also occurs in women during menopause and increases the risk of CVD,^29^ and lipid abnormalities have been directly related with AS.^30,31^ We herein proved that serum lipid levels were elevated by bilateral ovariectomy in ApoE^−/-^ mice in the absence of estrogen. Despite existing side effects, estrogen supplementation is still the main method for treating perimenopausal syndrome.^32,33^ Perimenopausal supplementation of estrogen can improve lipid metabolism^34^ and inhibit AS progression^35–37^ . We found that estrogen supplementation restored the serum lipid levels of ovariectomized ApoE^−/-^ mice to those of ApoE^−/-^ mice without receiving ovariectomy even after HFD feeding, suggesting that estrogen had a great influence on the lipid metabolism in females.

Hyperlipidemia during menopause injures the liver, which thus inhibits lipid metabolism and transport.^38^ Estrogen supplementation can alleviate the lesion of AS^39^ and liver lipid accumulation^40^ during perimenopause.^41^ After being consumed, lipids are mainly metabolized in the liver and mainly absorbed in the intestine.^42,43^ So, we then studied whether the lack of estrogen during menopause exerted identical effects in liver. We checked the changes of lipid-related enzymes during menopause with the occurrence of AS. The enzymes involved in lipid metabolism (especially in lipid biosynthesis, including LPCAT3, FASN, FDPS, Hmgcr, Hmgcs, SREBF, and SREBP) and transport (including ABCG5, ABCG8, ABCA1, ACAT1, LCAT, LDLR, LXR and SR-B1) significantly changed in the liver and intestine. Hence, estrogen deficiency during menopause impaired hepatic and intestinal functions related to lipid metabolism and transport, which may increase plasma lipid levels and accelerate menopausal AS progression^38,39^

Next, we investigated whether estrogen deficiency changed the lipid metabolomics in HFD-fed ApoE^−/-^ mice and whether the changes can be restored by estrogen supplementation. We have focussed on the changes of plasma lipid metabolites closely related with the enzymes mentioned above.^44,45^ Plasma CE, TG, phospholipids, and other types of lipids (including free fatty acids, acylcarnitine, sphingomyelins, and ceramides) significantly changed in different groups. Ovariectomy in combination with HFD markedly raised the levels of major plasma lipid metabolites, which were decreased by estrogen supplementation to be close to those of HFD-fed ApoE^−/-^ mice without receiving ovariectomy. Therefore, during menopause, estrogen loss may play a more important role than HFD in the progression of AS, which requires further validation.

The gut microbiota is involved in the regulation of lipids, especially in diseases associated with dyslipidemia, such as obesity^46,47^ and AS.^48,49^ Some specific gut microbiotas play key roles in the regulation of certain lipid metabolism-related enzymes.^50^ The link between lipid metabolomics changes and AS progression caused by estrogen decrease during menopause may be attributed to variations of the gut microbiota. In fact, menopause or AS has been significantly related with the gut microbiota.^51^ Jonsson et al. reported that the gut microbiota affected the progression of AS mainly through harmful local or deep inflammatory reactions caused by intestinal infections exacerbating AS plaque formation or causing plaque rupture, influence on the metabolism of cholesterol and lipids, and formation of specific substances.^12^ Herein, we studied whether estrogen deficiency and supplementation specifically changed the gut microbiota during menopause by detecting their compositions. Our results revealed specific changes of the gut microbiota in the feces of ovariectomized ApoE^−/-^ mice with or without estrogen supplementation. The gut microbiota species (OTUs) in ovariectomized ApoE^−/-^ mice had the lowest abundances among those of all groups, which were recovered by estrogen supplementation to be close to those of HFD-fed ApoE^−/-^ mice and even normally fed C57BL/6 mice. The relative abundances changed similarly at the phylum, family, or genus level. Taken together, estrogen exerted remarkable regulatory effects on the gut microbiota.

Abnormal diet leads to changes in gut microbiota, and the correlation between diet-induced changes in gut microbiota and changes in metabolites has been confirmed.^52^ Regulating gut microbiota may be a potential anti-hypercholesterolemia and hyperlipidemia therapy.^53^ Aggravated AS injury caused by estrogen loss during menopause has been strongly related with the gut microbiota^29^ or dyslipidemia.^9^ In order to further strengthen the relationship between changes in gut microbiota and dyslipidemia, as well as between the changes in gut microbiota and the process postmenopausal AS, we used fecal microbiota transplantation to intervene in gut microbiota in ovariectomized mice, and observed whether the interference of gut microbiota affects blood lipids, lipid metabolism and AS in postmenopausal stage. We think that the aggravation of AS caused by the absence of estrogen during menopause and high plasma cholesterol levels are inevitably associated with changes of the gut microbiota. Our results supplied the direct evidence that intervention in gut microbiota is sufficient to improve dyslipidemia, regulate lipid metabolism and reduce the symptoms of atherosclerosis in postmenopausal mice. We statistically analyzed the correlations between the gut microbiota and lipid metabolites in postmenopausal mice. As we expected, some specific gut microbiota and lipid metabolites showed significant negative or positive correlation.

In conclusion, we herein reported for the first time that during the progression of AS in perimenopausal mice, the specific changes of the gut microbiota were accompanied by the variations of plasma lipid metabolites. Furthermore, analyzing the correlation between the gut microbiota and lipid metabolomics indicated that the beneficial regulatory effects of the gut microbiota during menopause may reduce the risk of perimenopausal CVD by mitigating lipid metabolism disorders. In addition, estrogen supplementation significantly suppressed menopausal AS progression, hypercholesterolemia, and lipid metabolism disorders, also obviously regulating the gut microbiota. Moreover, intervention in gut microbiota by fecal microbiota transplantation is sufficient to improve blood lipids, AS symptoms, and lipid metabolism disorders in postmenopausal mice. Estrogen supplementation during menopause may delay the progression of AS and correct lipid metabolism disorders by regulating the gut microbiota. Notably, the findings provide theoretical support for estrogen replacement therapy, and detailed experimental evidence regarding the gut microbiota and lipid metabolism for understanding perimenopausal syndrome.

### 3.1. Limitations of study

Firstly, the correlations of estrogen deficiency and supplementation with the gut microbiota and lipid metabolites were not validated for normally fed ApoE^−/-^ mice. Secondly, the composition of the fecal gut microbiota is more like that of the large intestine, but the expressions of lipid-related genes in our study were only detected in the small intestine responsible for lipid absorption and transport. Further in-depth studies are needed to clarify the similarities and differences of genes related to lipid absorption and transport between large and small intestines. Thirdly, human subjects with or without AS in premenopausal and postmenopausal periods were not tested.

## 4. Materials and methods

### 4.1. Animals experimental design

Eight-week-old female C57BL/6 and ApoE^−/-^ mice were purchased from Beijing Hua Fukang Biological Technology Co., Ltd. (China). Four individuals were housed in each cage, with free access to food and water. Sixteen ApoE^−/-^ mice received bilateral ovariectomy and 90 days of HFD (including 0.3% cholesterol and 20% pork fat; Beijing Hua Fukang Biological Technology Co., Ltd., China). Eight ApoE^−/-^ mice were subjected to sham operation (needle threading, without ovariectomy) and maintained on HFD for 90 days. Eight C57BL/6 mice, which were used as a control group, were given sham operation, and maintained on a normal diet. Eight of the ApoE^−/-^ mice undergoing bilateral ovariectomy were intragastrical administrated with estrogen (0.13 mg/kg β-estradiol; Sigma-Aldrich, USA) daily for 90 days. Other mice were intragastrical administered with sterile carboxymethyl cellulose sodium (1%) daily.

In another independent experiment, twenty ApoE^−/-^ mice received bilateral ovariectomy and HFD for 90 days. These twenty mice also received fecal microbiota transplantation (FMT) every 3-day pre time for 90 days. Five of them received feces from C57BL/6 mice with sham operation and normal diet, five of them received feces from ApoE^−/-^ mice with sham operation and HFD, five of them received feces from ApoE^−/-^ mice with bilateral ovariectomy and HFD, and the last five of them received feces from ApoE^−/-^ mice with bilateral ovariectomy, HFD, and estrogen supplementation. Before these mice received FMT, broad-spectrum antibiotic was added to the drinking water (vancomycin 0.5 g/L and cefixime1g/L) to suppress the intestinal flora for two weeks. After that, fresh feces of donor mice were collected every three days. There were three mice in each group of the donors, and all donor mice were treated (sham operation or bilateral ovariectomy, normal diet or HFD, and estrogen supplementation) on the same time as the mice (bilateral ovariectomy and HFD) receiving fecal microbiota transplantation to ensure the feces were fresh. After the feces of each donor mouse were collected separately, the feces of the same group of donor mice were mixed and put into one sterile tube. After collecting, the feces were mixed in sterile PBS (1 g feces/ml PBS) and centrifuged at 500 rpm/min for 5 minutes. The supernatant was intragastrical administered to the recipient mice (0.1 ml/10 g).

All experimental procedures were performed in accordance with the national and international guidelines and regulations, and approved by Nanjing University of Chinese Medicine Animal Care and Use Committee (approval number: ACU-40(20141226) and ACU-02(20200425)).

### 4.2. Serum lipid detection

Blood was collected from the inner canthus after 90 days of administration and left still at room temperature for over 30 min, from which serum was separated by centrifugation at 1500 rpm for 10 min. Afterward, 100 µL of serum was collected from every sample. The serum TG, TC, LDL, and HDL levels were tested using biochemical kits (Jiancheng Bioengineering Institute, Nanjing, China) by HITACHI 7020 Chemistry Analyzer.^54^

### 4.3. Histological examination

After blood collection, all the mice were anesthetized with isoflurane before sacrifice. The liver and aorta were separated and fixed in 4% paraformaldehyde or 2.5% glutaraldehyde for 12–24 h. The atherosclerotic lesions of the aorta were evaluated by oil red O staining^55^ of thoracic aortic root cross-sections,^56^ and by scanning electron microscopy of aortic root cross-sections,^57^ The lipid deposition damage of the liver was evaluated by oil red O staining, hematoxylin-eosin (HE) staining, and transmission electron microscopy of liver tissues.^58^ Different histological examination methods were briefly described as follows.

For oil red O staining, liver tissues were fixed in 4% paraformaldehyde for over 12 h and rinsed with deionized water for 1 h. Then, the tissues were dehydrated with saturated sucrose solution for over 12 h, OCT-embedded, and cut into 10 μm-thick sections with a freezing slicer. Then, the sections were washed with water and 70% ethanol solution, stained by oil red O staining solution, washed with 70% ethanol solution, mounted with glycerin-gelatin jelly, and observed and photographed under an optical microscope. In contrast, the thoracic aorta was only dehydrated, washed, stained by the same reagent, and photographed by using a digital camera.

For HE staining, the liver tissues were fixed in 4% paraformaldehyde, rinsed by tap water for 1 h, dehydrated with different concentrations of ethanol solutions (70%, 80%, 90%, 95%, and 100%), transparentized by using xylene for 30 min and immersed in paraffin at 65°C for 45 min to fill the interstitial space. The resulting paraffin block was then cut into 5 μm-thick sections by using a slicer, heated at 65°C for 30 min, immersed in xylene for 20 min and rehydrated with different concentrations of ethanol solutions (100%, 95%, 90%, 80%, and 70%) and tap water. Subsequently, the sections were stained with hematoxylin staining solution for 3 min, rinsed with tap water for 20 min, stained with eosin staining solution for 30 s, washed with tap water, dehydrated by different concentrations of ethanol solutions (70%, 80%, 90%, 95%, and 100%), transparentized by xylene, mounted with neutral resin and photographed under an optical microscope

For electron microscopic imaging, the thoracic aorta and liver were fixed in 2.5% glutaraldehyde overnight. Afterward, the samples were immersed in ultrapure water for 1 h, re-fixed with osmic acid for at least 4 h, and dehydrated with different concentrations of tertiary butanol solutions (30%, 50%, 70%, 80%, 90%, and 100%), from which the remaining liquids were removed by a supercritical extractor. Then, each sample was treated with a gold-plated instrument and placed in a conductive copper mesh to be observed and photographed by scanning electron microscopy or transmission electron microscopy.

### 4.4. RNA isolation and real time-polymerase chain reaction (RT-PCR) analysis

Trizol reagent (Takara, Beijing, China) was used to extract total RNA from the liver and intestine (jejunum) according to the instructions of the manufacturer, and cDNA was synthesized by using a reverse transcription kit (Takara, Beijing, China). Target gene and internal control were then amplified by qPCR with ABI7500 real-time PCR system (Life Technologies, USA) according to the manufacturer’s instructions (Takara, Beijing, China). The sense and antisense primers used to detect LPCAT3, FASN, FDPS, HMGC, HMGCS, SREBP, ABCG5, ABCG8, ABCA1, ACAT1, LCAT, LDLR, LXR and SR-B1 expressions are listed in Table 1. All primers were synthesized by Sangon Biotech (Shanghai) Co., Ltd. (China).

**Table 1.** Primers used in RT-PCR analysis

	Forward (5’-3’)	Reverse (5’-3’)
LPCAT3	GCTGCGGCTCATCTTCTCCATC	TGAGAGGCCCGTGAAGGTGTG
FASN	TGCCACCCACCGTCAGAAGG	GTTCTTGCTGCCGCCGTGAG
FDPS	GTGGGCTGGTGTGTAGAACTGC	CAGAGCGTCGTTGATGGCATCC
HMGCR	GCCGTCATTCCAGCCAAGGTG	TTTGCTGCGTGGGCGTTGTAG
HMGCS	CGACGTCCCACTCCAATTGATG	TGCTTCAGGTTCTGCTGCTGTG
SREBP	CTGGTGCTGCTGCTGCTCTG	TCTCGGGCGGTGCGTAGC
ABCG5	CTGAGTCCAGAGGGAGCCAGAG	CACGGTTGCTGACGCTGTAGG
ABCG8	CCAACTGCTGCCCAACCTGAC	GCTCGGCGATTACGTCTTCCAC
ABCA1	GCGGAAGTTTCTGCCCTCTGTG	TGCTGGGTCGGGAGATGAGATG
ACAT1	GCCAGCACACTGAACGATGGAG	TGGGGTCTACGGCAGCATCAG
LCAT	AGAAGCTGGCTGGCCTGGTAG	GCTGCCGCAGTAAGAAGTGGAG
LDLR	GAGGAACTGGCGGCTGAAGAAC	CCTGGCTTCGGCAAATGTGGAG
LXR	TGAGGGAGGAGTGTGTGCTGTC	TGGCAGGACTTGAGGAGGTGAG
SR-B1	TCCAGTTCCAGCCCTCCAAGTC	CATCACCGCCGCACCCAAG
β-actin	GGCACCACACCTTCTACAATG	GGGGTGTTGAAGGTCTCAAAC

### 4.5. Lipid metabolism

a. Sample preparation

Blood was collected from mice after 90 days of administration and left still at room temperature for over 30 min, from which plasma was separated by centrifugation at 1500 rpm for 10 min. Samples were prepared based on liquid–liquid MTBE extraction to analyze lipid metabolism. Briefly, plasma (20 μL) was added into a 1.5 mL centrifuge tube, mixed with 225 μL of ice-cold methanol solution and internal standard (lysoPE (17:1), SM (17:0) for positive ion mode and PE (17:0/17:0) for negative ion mode; concentration: about 5 μg/mL), and vortexed for 10 s. Next, 750 μL MTBE was added, and the mixture were shaken for 10 min at 4°C. After 188 μL of deionized water was added, the mixture was vortexed for 10 s and then centrifuged at 14,000 rpm at 4°C. Lipids in the upper (organic) phase were transferred to clean tubes and dried by a vacuum centrifuge. Finally, the upper phase lipids were reconstituted with 110 μL of methanol: toluene (9:1) for LC-MS.

b. Untargeted lipidomic analysis

To detect lipids, 2 μL aliquots of sample solution were injected into a reversed-phase Waters Acquity UPLC CSH C18 column (100 mm × 2.1 mm, 1.7 μm) maintained at 60°C for gradient elution. Mobile phase A was water: ACN (6:4), and mobile phase B was isopropanol: ACN (9:1), both containing 10 mM ammonium formate and 0.1% formic acid. The flow rate was 0.3 mL/min. The elution gradient was as follows: 0–4.0 min, 15% B; 4.0–5.0 min, 15–48% B; 5.0–22.0 min, 48%–82% B; 22.0–23.0 min, 82–99% B; 23.0–24.0 min, 99% B; 24.0–24.2 min, 99%–15% B; 24.2–30.0 min, 15% B.

The spray voltage was 3.5 kV in the positive ion mode and 3.0 kV in the negative ion mode. For both ion modes, the sheath gas, aux gas, capillary temperature, and heater temperature were maintained at 35, 15 (arbitrary units), 325°C and 300°C, respectively.

c. Data processing

After lipid annotation, a small-scale database was set up with lipid name, retention time and accurate mass/charge ratio (m/z). Raw data files acquired from Xcalibur 2.2 software (Thermo Scientific, USA) were converted to the ABF format using ABF converter (accessible at: http://www.reifycs.com/AbfConverter). For data processing, MS-DIAL (v. 2.78) software program was used. In this study, only a lipid feature defined as an m/z – retention time pair can be aligned for an identical lipid. The resulting output data tables of high-quality time-aligned lipids together with the corresponding retention time, m/z and peak area were subjected to further statistical analysis. The screening conditions for relevant lipid metabolites were *p* value of ≤0.05 in combination with fold change of ≥1.5 or ≤0.667. PCA and OPLS-DA were performed according to the lipid metabolites. A heat map of serum lipid metabolites was also derived from the original data of lipid metabolism.

### 4.6. Mouse microbiota and statistical analyses

a. Fecal sample processing and microbial DNA extraction

The fecal samples of mice were frozen immediately in liquid nitrogen prior to euthanasia and stored at −80°C under sterile conditions until analysis. Fecal genomic DNA was extracted from the fecal samples with QIAamp® DNA Stool Mini Kit (Qiagen, Hilden, Germany) according to the manufacturer’s protocol. The DNA concentration and purity were detected by Nanodrop 1000 spectrophotometer (Thermo Fisher, Waltham, MA), and the DNA integrity was tested by 0.8% agarose gel electrophoresis.

b. 16SrRNA gene library preparation and high-throughput sequencing

Bacterial genomic DNA was used as the template to amplify the V3–V4 hypervariable region of 16SrRNA gene with high-fidelity Phusion polymerase (M0530S; Thermo Fisher, USA), forward primer (5’-CCTACGGGNGGCWGCAG-3’) and reverse primer (5’-GACTACHVGGGTATCTAATCC-3’). The universal sequence of the illumina adapter were added to the 5’ ends of each primer. Each sample was amplified by three repeated PCR experiments. The PCR products were tested by agarose gel electrophoresis. Then the products from the same sample were pooled, and purified using Agencourt AMPure XP Kit (Beckman Coulter, CA, USA). Then, using primers with Index sequence, a specific tag sequence compatible with the Illumina platform was introduced through high-fidelity PCR to construct the final complete library structure. The amplified products were purified with Agencourt AMpure XP magnetic beads to obtain an original library of samples. The library quality was assessed with Qubit@2.0 Fluorometer (Thermo Scientific, USA) and Agilent Bioanalyzer 2100 system (USA). The V3-V4 hypervariable regions of the 16S rRNA gene were sequenced using Illumina MiSeq Sequencer, at least 5 M 2x250bp pair-end raw reads were generated.

c. Sequence quality control and microbial community analysis

Raw reads were quality-filtered and merged with the following steps. First, the raw reads at any site with an average quality score of <20 were truncated and the adapter sequence and reads with lengths of <100 were removed by TrimGalore. Second, the paired reads were merged into tags by using Fast Length Adjustment of Short reads (FLASH, v1.2.11). The reads with ambiguous base (N base) and homopolymer of >6 bp were removed by Mothur. Third, the reads with low complexity were removed by using USEARCH to obtain clean reads for further bioinformatics analysis. The remaining reads were chimera-checked according to the gold.fa database (http://drive5.com/uchime/gold.fa) and clustered into OTUs by UPARSE with 97% similarity cutoff. Bioinformatics analysis was performed by Genesky Biotechnology Inc. (Shanghai, China).

All OTUs were classified based on Ribosomal Database Project. Alpha-diversities indicating within-sample richness were analyzed by Mothur. Sample tree cluster by Bray-Curtis distance matrix, unweighted pair-group method with arithmetic means and Jaccard principal coordinate analysis based on OTUs were conducted by R Project (Vegan package, V3.3.1). Redundancy was analyzed by Canoco for Windows 4.5 (Microcomputer Power, NY, USA) and assessed by MCPP with 499 random permutations. Linear discriminant effect size analysis was carried out to identify the microorganism features distinguishing fecal microbiota specifically for biomarker discovery. The two independent sample t-test and Mann – Whitney U test was performed to detect the significant differences of abundances among taxa.

### 4.7 Statistical analysis and correlation analysis

Data were represented as mean ± standard error of mean (mean ± SEM) if not indicated in another way. The four groups were compared using the Kruskal-Wallis test with the Dunn’s multiple comparisons test. The Mann–Whitney test was used to compare the differences of two groups. Differences were considered significant when *p* < .05. All statistical analyses were conducted with SPSS 23.0 for Windows (SPSS Inc., USA)

The correlations between the high-throughput 16SrRNA sequencing results of gut microbiota and plasma lipid metabolites were studied by analyzing those between species and environmental factors. Relevant original files, including Supplementary Table 3 and Table 4, were uploaded to the data analysis server (available at: http://cloud.geneskybiotech.com/login.html). The correlation coefficients (Pearson correlation coefficient) between the selected species and environmental factors were calculated. Finally, the correlation matrix was visualized through a heat map.

## Supplementary Material

Supplemental Material
